# The effect of trimethylamine N-oxide on the metabolism of visceral white adipose tissue in spontaneously hypertensive rat

**DOI:** 10.1080/21623945.2022.2104783

**Published:** 2022-08-17

**Authors:** Guo-Dong He, Xiao-Cong Liu, Xing-Hua Hou, Ying-Qing Feng

**Affiliations:** a School of Medicine, South China University of Technology, Guangzhou, 510006, China; b Department of Cardiology, Guangdong Cardiovascular Institute, Guangdong Provincial People’s Hospital, Guangdong Academy of Medical Sciences, Guangzhou, 510080, China; c Research Department of Medical Sciences, Guangdong Provincial People’s Hospital, Guangdong Academy of Medical Sciences, Guangzhou, 510080, China

**Keywords:** Trimethylamine N-oxide, visceral white adipose tissue, hypertension, fatty acids, phagosome, lysosome

## Abstract

Strong links have been reported among trimethylamine N-oxide (TMAO), visceral white adipose tissue (vWAT), and cardiometabolic diseases. However, the effects of TMAO on vWAT in hypertension remained incompletely explored. The impact of a chronic 22-week-long treatment with 1 g/L TMAO on vWAT, and its transcriptional and metabolic changes in spontaneously hypertensive rats (SHRs) were evaluated by serum cytokine measurements, histological analysis, fatty acid determinations, and co-expression network analyses. TMAO increased the serum interleukin-6 levels and insulin secretion in SHRs. The adipocyte size was diminished in the SHR 1 g/L TMAO group. In addition, one kind of monounsaturated fatty acids (cis-15-tetracosenoate) and four kinds of polyunsaturated fatty acids (cis-11,14,17-eicosatrienoic acid, docosatetraenoate, docosapentaenoate n-3, and docosapentaenoate n-6) were elevated by TMAO treatment. Three co-expression modules significantly related to TMAO treatment were identified and pathway enrichment analyses indicated that phagosome, lysosome, fatty acid metabolism, valine, leucine, and isoleucine degradation and metabolic pathways were the most significantly altered biological pathways. This study shed new light on the metabolic roles of TMAO on the vWAT of SHRs. TMAO regulated the metabolic status of vWAT, including reduced lipogenesis and an improved specific fatty acid composition. The mechanisms underlying these effects likely involve phagosome and lysosome pathways.

## Introduction

The gut microbiota represented a key intermediary between diet and host physiology, contributing to cardiometabolic diseases and associated hypertension [1,2]. However, specific cellular and molecular mechanisms by which gut microbial metabolites promote cardiometabolic diseases remain incompletely explored. Recently, multiple epidemiological and animal studies have indicated that elevated trimethylamine N-oxide concentrations are closely linked to the development of cardiometabolic diseases, such as insulin resistance and atherosclerosis [3–6].

Trimethylamine N-oxide (TMAO), an organic gut microbiota-derived metabolite mainly originating from dietary choline and L-carnitine, plays a prominent role in mechanistic links to the pathogenesis of atherosclerotic heart disease [7]. In addition to promoting atherosclerosis, TMAO has been correlated with prolonging and exacerbating the hypertensive status induced by angiotensin II in rats [8,9]. However, several experimental and clinical studies have described the circulatory system’s negative impact on TMAO [10,11]. Furthermore, TMAO supplementation slows aortic lesion formation in a mouse model [12] and exerts a beneficial effect on heart failure [13] by reducing the diastolic dysfunction in the pressure-overloaded hearts of hypertensive rats [14].

White adipose tissue (WAT), a heterogeneous organ composed of mature adipocytes, adipocyte progenitor cells, endothelial cells, fibroblasts, and diverse types of immune cells, functions in energy storage and as an endocrine organ [15]. Bioactive molecules, such as free fatty acids, leptin, and pro-inflammatory cytokines, can be released into circulation by WAT. These molecules may contribute to cardiometabolic disorder [16,17]. The TMAO-producing enzyme Fmo3 acts as a negative regulator of programmed WAT [18]. In addition, accumulated evidence indicates that TMAO behaves similarly to hormones in adipose tissue [18,19].

Owing to the strong links between TMAO, WAT, and cardiometabolic diseases, we hypothesized that TMAO compromises WAT’s ability to maintain metabolic homoeostasis in cardiometabolic diseases. This study evaluated the impact of a chronic 22-week-long treatment with TMAO on visceral WAT (vWAT), and its transcriptional and metabolic changes, in spontaneously hypertensive rats (SHRs).

## Materials and methods

### Animal treatments

Male SHRs and normal Wistar-Kyoto (WKY) rats were provided by Beijing Vital River Laboratory Animal Technology Co. Ltd. The rats were housed at Forervegen’s Experimental Animal Center (Guangzhou, China) and were kept in groups of three animals in polypropylene cages under a 12-h light/12-h dark cycle at 22–23°C and humidity of 45%–55%. They were provided a standard laboratory diet and water ad libitum. Seven- to eight-week-old SHRs (n = 12) were randomly assigned to a water group, either drinking tap water or water containing 1 g/L TMAO (product number: T1362, Tokyo Chemical Industry, Japan). The dose of 1 g/L of TMAO was selected to increase the plasma TMAO by 9–18-fold to mimic physiological high-dose TMAO concentrations based on previous study results [14]. After 22 weeks of treatment with water or TMAO, rats were maintained in metabolism cages for 2 days to evaluate their 24-h food balance. Blood glucose levels of rats were measured after fasting over-night. Fasting serum was collected by cardiac puncture, and vWAT was sampled from each abdominal cavity.

All animal care and experimental protocols were in accordance with the National Institutes of Health Guide for the Care and Use of Laboratory Animals and were approved by the Research Ethics Committee of Guangdong Provincial People’s Hospital.

### Evaluation of TMAO concentration

The blood plasma concentration of TMAO was determined using liquid chromatography coupled with triple-quadrupole mass spectrometry as previously described [8]. The ion transitions were m/z 76.1 > 58.0 for TMAO and m/z 85.1 > 66.0 for TMAO-D9. The calibration curve range was 1–5,000 ng/mL for TMAO.

### Measurements of circulating cytokines

Luminex tests were used to determine serum cytokines (Luminex MAGPIX, Luminex Corp.) using appropriate kits (Millipore) in accordance with the manufacturer’s guidelines. We measured and analysed six cytokines, interleukin (IL)-6, IL-1β, tumour necrosis factor-α (TNF-α), macrophage chemoattractant protein-1 (MCP-1), insulin, and leptin. Assays were analysed on Luminex 200 (Luminex Corp.).

### Haematoxylin-eosin (HE) and Masson staining of vWAT

The paraffin sections of vWAT were subjected to HE and Masson staining using HE staining (abs9217, Absin) and Masson Trichrome Staining (abs9348, Absin) kits following the manufacturer’s specifications. The images were analysed using ImageJ software (ImageJ v.1.8.0).

### Determination of the adipose tissue medium- and long-chain fatty acids (MLCFAs)

For fatty acids extraction, 60 mg of vWAT was transferred to a centrifuging tube. After adding 1 mL chloroform/methanol solution, each sample was ultrasonicated for 30 min, centrifuged at 2,000 rpm for 10 min, followed by supernatant removal. Then, 2 mL 1% sulphuric acid/methanol solution was added for 0.5-h methyl esterification in a water bath at 80°C. For extraction, 1 mL of n-hexane was added, and for washing, 5 mL of water was added. A total of 500 μL extract supernatant was mixed with 25 μL of internal standard (methyl nonadecanoate) before injection. For standard preparation, the mixed standard solution of 40 components of methyl esterified fatty acids (Sigma-Aldrich) was used as a reference standard to identify the fatty acids as previously described [20]. Ten mixed standard concentration gradients of 0.5, 1, 5, 10, 25, 50, 100, 250, 500, and 1,000 mg/L were prepared, in which the concentration of each component accounted for 2% and 4% of the total concentration of two gradients, with 30 and 10 components accounting for 2% and 4%, respectively. An Agilent DB-WAX capillary column (30 m × 0.25 mm ID × 0.25 µm) was used to separate the methyl esterified fatty acids. The temperature was initially set to 50°C for 3 min and then escalated at a rate of 10°C/min to 220°C for 20 min. Helium was used as the carrier gas, and the carrier gas velocity was set at 1.0 mL/min. The analysis was performed using an Agilent 7890A/5975C gas chromatography-mass spectrometer. Agilent ChemStation software was used to determine the retention times and areas of chromatographic peaks. Each sample’s MLCFA content was determined by graphing the curve.

### RNA preparation, sequencing, and analysis

RNA of vWAT was isolated using the TRIzol reagent/chloroform isolation method. The full-length complementary DNAs and RNA-seq libraries were prepared using SMART technology (Clontech) [21]. RNA sequencing was carried out using an illuminated Nova-Seq 6000 System with the assistance of Guangzhou Epibiotek Co., Ltd. The GENCODE database was utilized to annotate mRNAs [22]. RNA reads were counted using feature Counts [23]. To normalize gene expression counts for the sequence, the fragments per kilobase of transcript per million fragments mapped (FPKM) approach was used. A principal component analysis (PCA) was used to evaluate variables within the three groups. Data were transformed into log2 scale and plotted using the plotPCA function in R v.3.5.2.

### Differential expression analysis

Expression levels were estimated on the basis of the high-throughput sequences by differential expression analysis. The raw counts from the sequence datasets were used as in input in the ‘DESeq2’ R package (DESeq2 1.16.1) [24] to obtain differentially expressed genes based on log2 fold change ≥ |1.0| at a statistical significance of adjust p-value < 0.05. The R heatmap and ggplot2 package were applied to construct the heatmap.

### Co-expression networks of mRNAs

First, we eliminated genes with expression levels lower than 1 FPKM in at least 90% of the samples. The remaining genes having a standard deviation larger than 0.2 were further maintained for a downstream weighted gene co-expression network analysis (WGCNA). Modules of co-expression networks were determined using the ‘WGCNA’ package in R as previously described [25]. Briefly, a suitable soft threshold power was determined using the scale-free topology requirement. Then, the weighted adjacency matrix was generated. Correlations and adjacencies were converted into a matrix of topological overlap (TOM), after which the equivalent dissimilarity was calculated (1-TOM). Next, genes were clustered hierarchically using 1-TOM as the distance metric. The dynamic tree cut technique with default parameters was used to discover modules. For each module, Kyoto Encyclopaedia of Genes and Genomes (KEGG) enrichments were conducted to understand the enriched functions using R package ‘clusterprofile’. The PCA of each gene module evaluated module eigengenes (MEs) as the major components, and within a specific module, the expression patterns of all the genes might be summed into a single characteristic expression profile. Significant associations were defined significantly if the module had a p-value < 0.05 and an eigengene-trait absolute correlation > 0.7.

### Statistical analysis

Undetectable cytokine concentrations, where the analyte concentration was less than the lower limit of quantification (LLOQ), were replaced with LLOQ/√2. The means ± standard deviations (SDs) of ongoing parameters were provided. Student’s t-tests or Mann-Whitney U tests were used to assess variations between two groups. To investigate the significant variations between three or more groups, a one-way analysis of variance with Tukey’s multiple comparisons test or Kruskal-Wallis with multiple comparison testing was used. A two-tailed p-value of < 0.05 was defined as statistically significant. All the statistical analyses were conducted using R programming language version 4.0.

## Results

### TMAO regulated the serum levels of cytokines and insulin in SHRs

After 22 weeks of treatment with TMAO, the TMAO plasma level of rats in the SHR 1 g/L TMAO group were significantly higher than in the SHR-water and WYK groups (Table 1). However, there were no significant differences in food intakes and body weights between groups (Table 1). This indicated that TMAO had no influence on food balance.

**Table 1.** TMAO regulated the serum levels of IL-6 and insulin in SHRs.

Variables	WKY Group	SHR-Water Group	SHR-TMAO Grop	P Value (by ANOVA)
Body mass and food intake				
Body mass, g	349.18 ± 21.15	335.41 ± 23.45	357.18 ± 17.38	0.23
24 h food intake, g	15.65 ± 3.24	13.47 ± 1.40	12.51 ± 1.86	0.09
TMAO				
Plasma TMAO, ng/ml	102.48 ± 44.14	144.72 ± 25.27	4031.03 ± 1035.35*†	< 0.01
Circulating cytokines				
IL-6, pg/ml	17.29 ± 0.00	17.29 ± 2.29	41.79 ± 19.60*†	< 0.01
IL-1β, pg/ml	9.04 ± 8.14	5.18 ± 6.75	2.04 ± 0.83	0.14
TNF-α, pg/ml	1.49 ± 0.72	0.81 ± 0.27	1.17 ± 0.52	0.17
MCP-1, pg/ml	169.22 ± 70.01	122.09 ± 63.35	180.91 ± 31.35	0.15
leptin, pg/ml	6436.17 ± 755.63	2424.40 ± 709.16*	1957.83 ± 1110.89*	< 0.01
Insulin and FBG				
Insulin, pg/ml	3060.67 ± 1268.03	6988.40 ± 2304.11*	22729.17 ± 19264.28*†	0.01
FBG, mmol/l	4.28 ± 0.28	4.94 ± 0.43*	4.68 ± 0.18	0.02

Values are means ± SD. Data are for Wistar-Kyoto (WKY) rats (n = 6), spontaneously hypertensive rats (SHRs) treated with water (SHR-Water; n = 6), and SHRs treated with 1 g/L trimethylamine oxide (TMAO) in drinking water (SHR-TMAO; n = 6). One-way ANOVA followed by a Tukey’s post hoc test was used. *P < 0.05 vs. the WKY group; †P < 0.05 vs. the SHR-Water group by a Tukey’s post hoc test. FBG: fasting blood glucose.

Because circulating TMAO is considered to be associated with increases in inflammatory cytokines [26], we measured five serum inflammatory cytokines, IL-6, IL-1β, TNF-α, MCP-1, and leptin, to ascertain the effects of chronic TMAO treatment on vWAT in SHRs. With the 1 g/L TMAO treatment, only the IL-6 serum level was markedly elevated, whereas other cytokines did not show statistical differences (Table 1). However, inflammatory responses might still not be confirmed because IL-6 had context-dependent pro-and anti-inflammatory properties [27]. In addition, TMAO is positively associated with cardio-metabolic risk factors, such as insulin resistance and metabolic syndrome [28], and the SHR 1 g/L TMAO group showed dramatically elevated serum insulin levels in this study (Table 1). However, TMAO-treated SHRs demonstrated mild decreases in fasting blood glucose levels compared with the SHRs groups, but it was not significant (P > 0.05).

Owing to the results of serum cytokine and insulin levels, we focused on the effects of TMAO on the metabolism of vWAT in SHRs because IL-6 and insulin regulate adipose tissue, but they had opposite effects [29,30].

### TMAO regulated the vWAT size in SHRs

**Figure 1.**
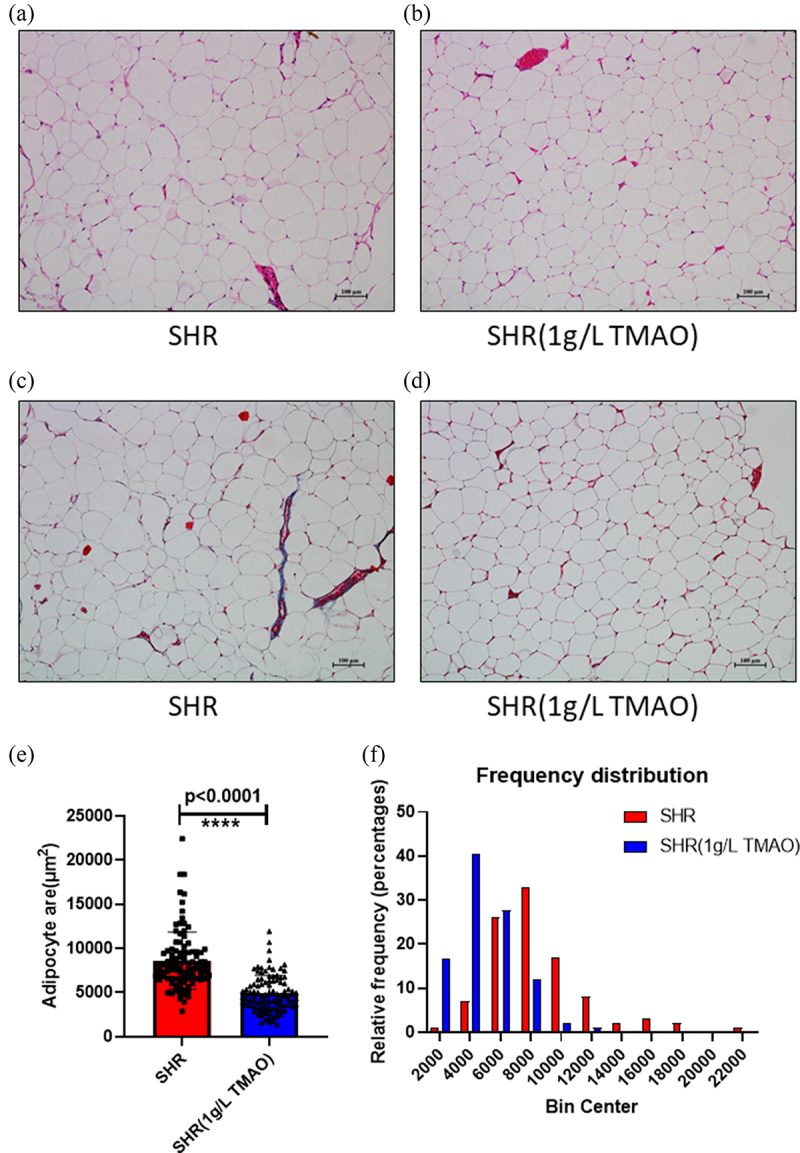
Adipose histology and quantification. a, b: Representative images of haematoxylin and eosin stain from visceral white adipose tissue in the spontaneously hypertensive rat (SHR) group (a) and the SHR 1 g/L TMAO group (b). c, d: Representative images of Masson stain from visceral white adipose tissue in SHR (c) and SHR 1 g/L TMAO (d) groups. e: Adipocyte area. The results are expressed as means ± SDs. The differences between the two groups were statistically analysed using Student’s t-tests. The data represent statistical significance at ****p < 0.0001. f: Histogram of adipocyte count distribution of specified adipocyte area; 100 adipocytes per mouse were used for quantitative evaluation.

To evaluate the effects of elevated levels of IL-6 and insulin on vWAT mediated by TMAO, we observed the morphology of vWAT using HE and Masson staining. Adipocyte size was assessed using HE staining to determine whether adipocyte hypertrophy occurred after a long TMAO treatment period (Figure 1aandb).

The size of adipocytes was diminished in the SHR 1 g/L TMAO group. Furthermore, Masson staining was used to examine fibrosis in vWAT caused by collagen deposition (Figure 1candd). No evidence of adipose fibrosis was found. Thus, the effects of adipocyte volume diminishment after TMAO treatment might be associated with increased IL-6 secretion.

### Identification and quantification of MLCFAs

Our results previously showed that the TMAO treatment induces IL-6 secretion and diminishes vWAT. Furthermore, owing to the well-known stimulatory effects of IL-6 on lipolysis, as well as fatty acid oxidation [31], we applied 40 fatty-acid methyl ester standards to a gas chromatography-tandem mass spectrometer analysis to further ascertain the impact of TMAO on fatty acid metabolism in vWAT.

A total of 34 different kinds of fatty acids have been identified and quantified in the vWAT samples (Table 2), of which six fatty acids were undetected in all the samples. As shown in Table S1, the standard curve, linearity, and correlation coefficient (R^2^) of each component were in the selected concentration range. The linearity and correlation coefficient for each component was > 0.999 (Table S1).

**Table 2.** The composition of medium- and long- chain fatty acids found in vWAT from WKY, SHR-Water, and SHR-1 g/L TMAO groups.

		Mean ± SD (ug/mL)			*p*-value
Component	MLCFAs	WKY	SHR-Water	SHR-1 g/L TMAO	(by ANOVA)
**SFA**					
Total SFA	/	52117.910 ± 10630.584	55661.060 ± 3004.829	54037.027 ± 2482.367	NS
C10:0	decanoate	32.931 ± 7.08	30.315 ± 3.000	27.762 ± 3.669	NS
C11:0	undecanoate	1.615 ± 0.244	1.218 ± 0.242*	1.177 ± 0.187*	0.008
C12:0	dodecanoate	143.254 ± 48.983	157.897 ± 23.382	131.426 ± 10.765	NS
C14:0	myristate	2403.795 ± 735.672	2939.138 ± 400.367	2554.393 ± 241.84	NS
C15:0	pentadecanoate	604.943 ± 95.525	407.972 ± 69.976*	417.751 ± 61.53*	<0.001
C16:0	palmitate	43348.974 ± 8945.043	46846.530 ± 2658.387	45803.567 ± 2248.655	NS
C17:0	heptadecanoate	627.358 ± 93.689	420.187 ± 43.349*	420.692 ± 55.311*	<0.001
C18:0	stearate	4340.754 ± 903.28	4438.812 ± 82.326	4238.007 ± 274.481	NS
C20:0	arachidate	272.060 ± 52.552	201.562 ± 24.866*	187.234 ± 25.007*	0.002
C21:0	heneicosanoate	16.617 ± 3.87	10.366 ± 1.993*	10.716 ± 1.458*	0.001
C22:0	behenate	257.650 ± 39.618	160.821 ± 33.310*	203.689 ± 35.711*	0.001
C23:0	tricosanoate	14.549 ± 3.499	9.106 ± 2.167*	8.168 ± 1.321*	0.001
C24:0	tetracosanoate	53.410 ± 11.095	37.135 ± 8.945*	32.445 ± 7.151*	0.003
**MUFA**					
Total MUFA	/	99163.218 ± 21219.853	106566.155 ± 6668.756	102210.392 ± 5432.69	NS
C14:1N5	myristoleate	82.945 ± 36.141	142.266 ± 39.530	112.270 ± 39.12	NS
C15:1N5	cis-10-pentadecenoate	24453.243 ± 5319.744	26613.658 ± 1535.105	26002.833 ± 1300.146	NS
C16:1N7	palmitoleate	6618.981 ± 2973.065	10,172.508 ± 1818.967	9027.605 ± 1995.191	NS
C17:1N7	cis-10-heptadecenoate	245.269 ± 50.836	243.384 ± 31.908	240.533 ± 19.507	NS
C18:1TN9	elaidate	59453.262 ± 11706.423	61168.845 ± 3659.755	58346.452 ± 2955.179	NS
C18:1N9	oleate	7105.363 ± 1558.791	7294.707 ± 549.152	7423.020 ± 674.394	NS
C20:1N9	cis-11-Eicosenoic acid	609.412 ± 98.469	492.531 ± 42.099*	500.322 ± 36.212*	0.012
C22:1N9	erucate	90.740 ± 24.714	88.875 ± 29.009	90.567 ± 24.441	NS
C24:1N9	cis-15-tetracosenoate	504.002 ± 78.387	349.381 ± 54.404*	466.791 ± 90.456†	0.008
**PUFA**					
Total PUFA	/	90744.451 ± 12068.822	71859.955 ± 6915.356	74212.841 ± 5411.218	0.003
C18:2N6	linoleate	82805.971 ± 11050.110	65731.671 ± 5846.682*	67139.866 ± 5244.781*	0.003
C18:3N6	γ-linolenate	172.574 ± 43.026	178.344 ± 41.249	211.010 ± 29.839	NS
C18:3N3	α-linolenate(ALA)	4165.654 ± 458.244	3153.924 ± 502.287*	3335.967 ± 196.782*	0.001
C20:2N6	cis-11,14-Eicosadienoic acid	541.499 ± 85.57	446.854 ± 80.936	539.826 ± 27.926	NS
C20:3N6	cis-8,11,14-Eicosatrienoic acid	253.191 ± 58.59	208.937 ± 38.409	279.975 ± 39.021	NS
C20:4N6	arachidonate	1628.711 ± 275.266	1327.785 ± 260.249	1590.469 ± 317.444	NS
C20:3N3	cis-11,14,17-Eicosatrienoic acid	35.540 ± 3.459	27.875 ± 4.903*	35.111 ± 5.219†	0.018
C20:5N3	cis-5,8,11,14,17-Eicosapentaenoic acid (EPA)	39.624 ± 8.377	26.102 ± 5.013*	24.896 ± 3.753*	0.001
C22:4N6	docosatetraenoate	465.513 ± 114.483	315.139 ± 80.688*	456.924 ± 120.188†	0.047
C22:5N6	docosapentaenoate n-6	54.818 ± 14.407	51.037 ± 15.106	82.354 ± 25.877*†	0.025
C22:5N3	docosapentaenoate n-3 (DPA)	517.826 ± 104.889	318.490 ± 87.286*	445.799 ± 103.421†	0.011
C22:6N3	cis-4,7,10,13,16,19-Docosahexaenoic acid (DHA)	63.530 ± 20.416	73.797 ± 23.163	70.643 ± 23.198	NS
**Undetectable**					
C22:2N6	cis-13,16-Docosadienoic acid	/	/	/	/
C4:0	butyrate	/	/	/	/
C6:0	hexanoate	/	/	/	/
C8:0	octanoate	/	/	/	/
C13:0	tridecanoate	/	/	/	/
C18:2TTN6	linolelaidate	/	/	/	/

Values are means ± SD. Data are for Wistar-Kyoto (WKY) rats (n = 6), spontaneously hypertensive rats (SHRs) treated with water (SHR-Water; n = 6), and SHRs treated with trimethylamine oxide (TMAO) in drinking water (SHR-TMAO; n = 6). One-way ANOVA followed by a Tukey’s post hoc test was used. TMA, trimethylamine; NS, no significant differences among groups by ANOVA. *P < 0.05 vs. the WKY group; †P < 0.05 vs. the SHR-Water group by a Tukey’s post hoc test

There were no noticeable distinctions between the concentrations of total saturated fatty acid (SFA) and total monounsaturated fatty acid (MUFA) in the three groups, but the total polyunsaturated fatty acid (PUFA) level in the WKY group was higher than in the other groups. When compared with levels in WKY rats, 16 MLCFAs obviously decreased (*p* < 0.05) in SHRs treated with water, including eight SFAs (undecanoate, pentadecanoate, heptadecanoate, arachidonate, heneicosanoate, behenate, tricostate, and tetracosanoate), two MUFAs (cis-11-eicosenoic acid ester and cis-15-tetracosenoate), and six PUFAs [linoleate, linolenate, cis-11,14,17-ricosatrienoic acid ester, cis-5,8,11,14,17-ricosapentaenoic acid ester, docosatetraenoate, and docosapentaenoic (C22:5N3)]. Among them, one MUFA (cis-15-tetracosenoate) and four PUFAs [cis-11,14,17-ricosatrienoic acid, docosatetraenoate, docosapentaenoate n-3 (C22:5N3), and docosapentaenoate n-6 (C22:5N6)] were elevated by the TMAO treatment. In this study, our results showed that TMAO induced IL-6 secretion and regulated the composition of fatty acids, especially PUFAs, in vWAT.

### RNA sequencing and co-expression network analysis of vWAT

To further understand the experimental results, we continued exploring the possible mechanisms using RNA-seq.

**Figure 2.**
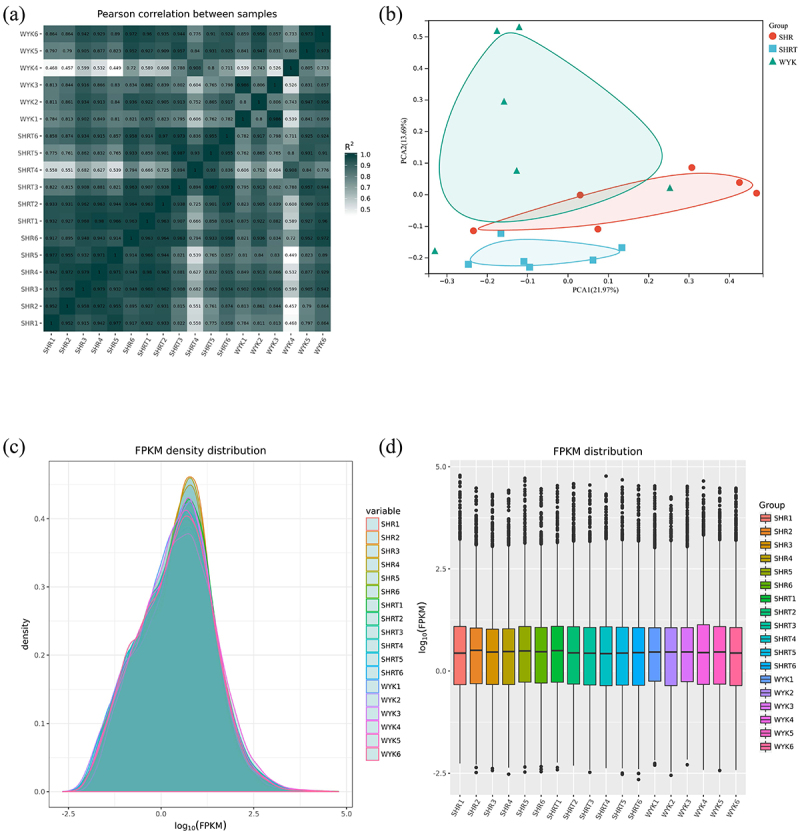
A: Pearson’s correlations among samples based on expression levels. B: Principal component analysis of 18 samples based on expression levels. C: Density plot displaying the gene density at different FPKM levels. D: Box plot of the FPKM distribution among the 18 samples. SHRW: white adipose tissue of spontaneously hypertensive rat, SHRWT: white adipose tissue of TMAO treated spontaneously hypertensive rat, WYKW: white adipose tissue of WYK.

**Figure 3.**
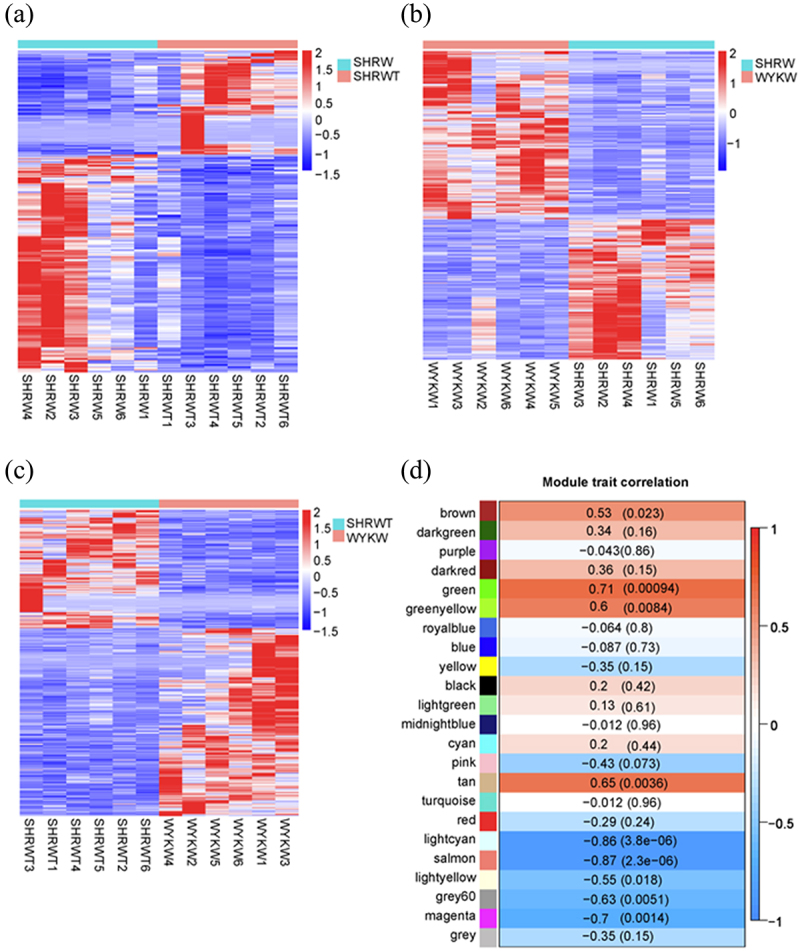
A-C: Hierarchical cluster analysis among the three groups. SHRW: white adipose tissue of spontaneously hypertensive rat, SHRWT: white adipose tissue of TMAO treated spontaneously hypertensive rat, WYKW: white adipose tissue of WYK. D: The correlations between each functional module and phenotype. Colours correspond to correlations. Positive correlations are shown by red, and negative correlations are shown by green. Additionally, correlation coefficients and P values are marked.

First, we confirmed the reliability of the RNA-seq data using a Pearson’s correlation analysis, principal component analysis, and the FPKM density distribution of the 18 samples (Figure 2a–). A high repeatability in the sequencing results was achieved in each group. In addition, a hierarchical cluster analysis indicated that the gene expression patterns in vWAT were distinguishable among the three groups (Figure 3a–). The top 25 up- and top 25 down-regulated mRNAs in the SHR group vs. the SHR 1 g/L TMAO group are shown in Table 3.

**Table 3.** The top 25 up and top 25 down regulated mRNAs in SHR group vs. SHR 1 g/L TMAO group.

gene_name	baseMean	log2FoldChange	pvalue	padj
Gsta3	7.04	23.25	7.69E-20	1.13E-15
Pax5	10.33	18.51	9.91E-14	7.29E-10
Clec4m	15.76	18.30	1.43E-10	4.72E-07
Marco	34.20	17.02	2.12E-05	0.005451
Nppa	22.72	16.34	1.22E-10	4.72E-07
Sptlc3	6.89	6.66	0.000133	0.012946
Prom2	94.81	6.53	1.99E-05	0.005451
Tnnt2	593.89	6.35	5.66E-05	0.008671
Pnoc	5.10	5.93	0.000257	0.016091
LOC103692785	5.12	4.78	2.18E-05	0.005451
Actg2	126.63	4.77	1.65E-07	0.000221
Cldn7	15.56	4.73	7.79E-05	0.00963
LOC103690054	11.97	4.22	3.01E-06	0.001578
Gstm3	69.53	4.15	0.000137	0.01303
LOC100912599	144.81	4.14	0.000307	0.01732
Tmc4	8.09	4.05	0.000783	0.025
Esrp1	12.50	4.00	0.001227	0.030941
AABR07015881.1	6.81	3.98	0.00227	0.041812
LOC100911422	35.43	3.73	3.17E-05	0.006959
Cd24	55.75	3.61	0.000194	0.015397
Krt20	5.62	3.56	0.001858	0.038065
Fam84a	9.26	3.53	0.001343	0.032033
Pls1	8.98	3.42	0.00238	0.04281
Crabp2	47.26	3.42	3.31E-05	0.00701
Fam50a	32.38	3.36	0.000847	0.025416
Zfp366	41.40	−2.22	2.52E-05	0.005981
Gan	8.35	−2.26	0.00146	0.033424
AABR07055864.2	10.81	−2.28	0.002134	0.040883
Setbp1	7.64	−2.32	0.002503	0.043924
Tmtc1	14.81	−2.35	0.000333	0.017717
Fat4	46.36	−2.35	7.59E-05	0.009611
Amer1	13.75	−2.36	0.000606	0.022419
LOC103693776	20.96	−2.39	0.000189	0.0153
Fosb	84.33	−2.42	0.002974	0.047383
Zc3h12c	8.59	−2.42	4.74E-05	0.008495
Marf1	23.64	−2.46	0.000936	0.02746
Slain1	5.85	−2.63	6.25E-05	0.00912
AABR07033887.1	30.59	−2.70	6.12E-05	0.00912
Cdhr1	6.51	−2.74	0.002628	0.044647
Mcc	15.59	−2.77	3.76E-05	0.007593
LOC103694908	7.59	−2.88	0.00073	0.024333
LOC100911672	30.07	−2.95	7.83E-05	0.00963
LOC100362814	4.67	−3.22	0.002716	0.04531
Scube3	4.45	−3.29	0.002403	0.042917
Eomes	4.43	−3.31	0.001883	0.03838
Rnf112	5.12	−3.67	0.002386	0.04281
Lrrn4	29.17	−3.74	0.001925	0.038857
LOC100910875	15.44	−4.05	0.000208	0.015523
AABR07054286.1	4.35	−4.26	0.000477	0.020284
AABR07000398.1	666.24	−7.26	7.46E-07	0.000522

To further identify pathways significantly related to the TMAO treatment on vWAT, a weighted gene co-expression network analysis was used to construct gene co-expression networks. We used an empirically derived soft threshold of 11 to stress the importance of strong correlations in network development, encapsulating a robust model suitable for scale-free topology (R^2^ > 0.80, Figure S1A). In total, 23 modules of mRNA expression were identified through a hierarchical clustering dendrogram (Figure S1B). Using a nominally significant cut-off (cor > 0.50, *p* < 0.05), nine module-trait relationships emerged (Figure 3d). The three highest correlations were detected using the module-trait association analysis for the green (cor = 0.71, *p* = 0.00094), light cyan (cor = −0.86, *p* = 3.8e^−06^), and salmon (cor = −0.87, *p* = 2.3e^−06^). Additionally, a module-module correlation analysis revealed that the green module was correlated with both light cyan (cor = −0.58, *p* = 0.012) and salmon (cor = −0.85, *p* = 7.2e^−06^) modules (Figure S1C).

Finally, the green, light cyan, and salmon gene-expression modules were selected for further investigation.

### Pathway enrichment analysis of co-expression modules

Because the green, light cyan, and salmon modules were strongly associated with TMAO therapy, and the gene co-expression modules were comprised of highly correlated genes, we performed a KEGG gene enrichment analysis to further investigate the biological functions of these gene modules.

**Figure 4.**
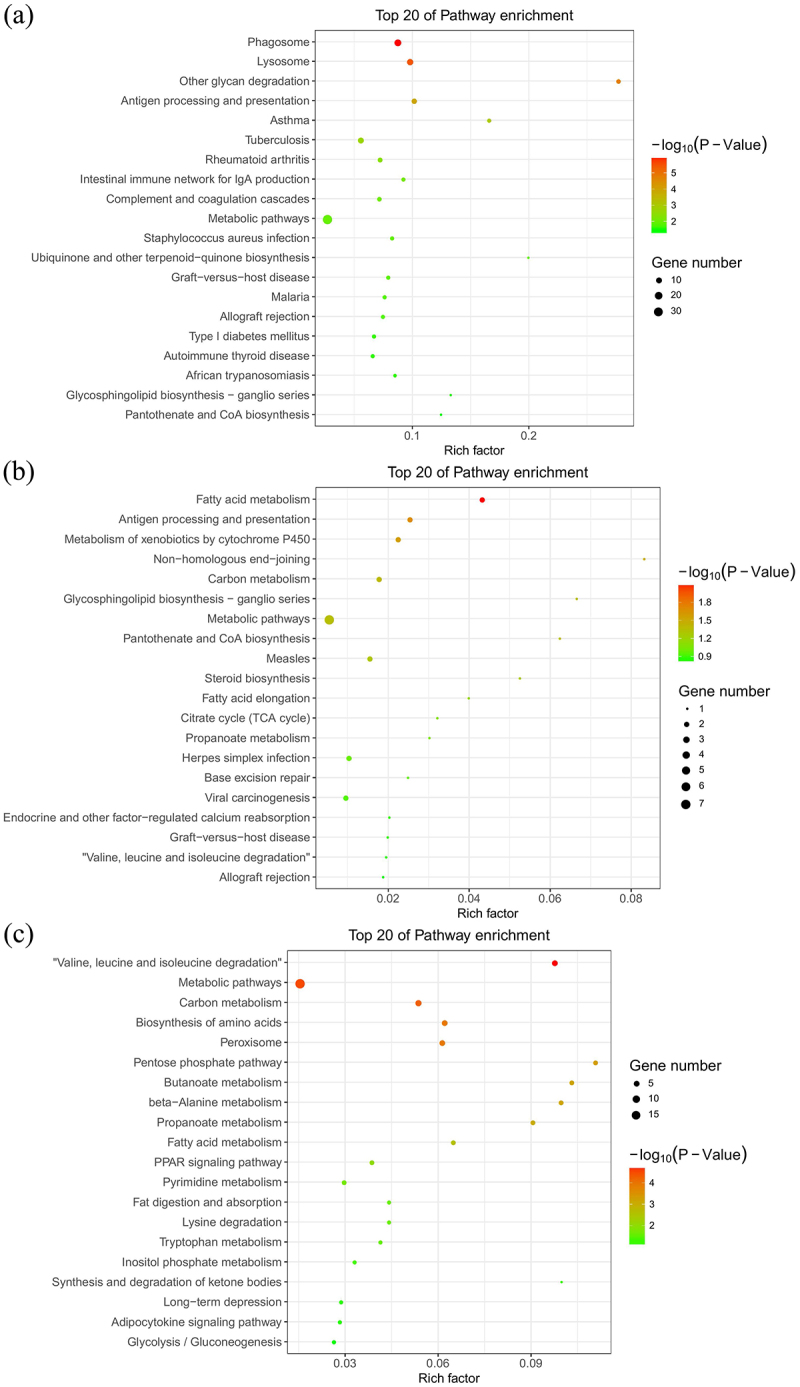
Kyoto encyclopaedia of genes and genomes pathway enrichment of genes in the green (a), light cyan (b), and salmon (c) modules generated by hierarchical clustering of WGCNA. The size and the colour intensity of a circle represented gene number and -log10 (P-value), respectively. Rich Factor was considered the ratio of the number of genes in a certain module to the total number of genes in the pathway.

The top 20 KEGG pathway terms were identified from genes in each module (Figure 4a–). Among the altered signalling pathways, the most significantly altered biological pathways included phagosome, lysosome, fatty acid metabolism, valine, leucine and isoleucine degradation, and metabolic pathways. The genes categorized for these pathways are listed in Table 4. *Lamp1* and *Ctss* played roles in both the phagosome and lysosome pathways. In addition, *Ehhadh* and *Aldh3a2* were shown to function in both fatty acid metabolism and valine, leucine and isoleucine degradation pathways.

**Table 4.** The gene name and enrichment levels categorized for phagosome, lysosome, fatty acid metabolism, and valine, leucine and isoleucine degradation.

PathwayTerm	Symbol	Description	FDR	Enrichment
Fatty acid metabolism	Ehhadh	enoyl-CoA, hydratase/3-hydroxyacyl CoA dehydrogenase	0.031	10.08
Fatty acid metabolism	Cpt2	carnitine palmitoyltransferase 2	0.29000	14.56
Fatty acid metabolism	Aldh3a2	aldehyde dehydrogenase 3 family, member A2	0.03100	10.08
Fatty acid metabolism	Acsl4	acyl-CoA synthetase long-chain family member 4	0.03100	10.08
Fatty acid metabolism	Acaa2	acetyl-CoA acyltransferase 2	0.29000	14.56
Lysosome	Smpd1	sphingomyelin phosphodiesterase 1, acid lysosomal	0.00041	4.94
Lysosome	Neu1	neuraminidase 1	0.00041	4.94
Lysosome	Man2b1	mannosidase, alpha, class 2B, member 1	0.00041	4.94
Lysosome	Lgmn	legumain	0.00041	4.94
Lysosome	Lamp1	lysosomal-associated membrane protein 1	0.00041	4.94
Lysosome	Hexa	hexosaminidase A	0.00041	4.94
Lysosome	Fuca1	fucosidase, alpha-L- 1, tissue	0.00041	4.94
Lysosome	Ctss	cathepsin S	0.00041	4.94
Lysosome	Ctsh	cathepsin H	0.00041	4.94
Lysosome	Ap1s2	adaptor-related protein complex 1, sigma 2 subunit	0.00041	4.94
Lysosome	Aga	aspartylglucosaminidase	0.00041	4.94
Lysosome	Acp5	acid phosphatase 5, tartrate resistant	0.00041	4.94
Phagosome	Tubb6	tubulin, beta 6 class V	0.00020	4.42
Phagosome	Tubb2a	tubulin, beta 2A class IIa	0.00020	4.42
Phagosome	Tuba1b	tubulin, alpha 1B	0.00020	4.42
Phagosome	Tap1	transporter 1, ATP-binding cassette, sub-family B (MDR/TAP)	0.00020	4.42
Phagosome	RT1-T24-4	RT1 class I, locus T24, gene 4	0.00020	4.42
Phagosome	RT1-Bb	RT1 class II, locus Bb	0.00020	4.42
Phagosome	RT1-Ba	RT1 class II, locus Ba	0.00020	4.42
Phagosome	RT1-A2	RT1 class Ia, locus A2	0.00020	4.42
Phagosome	Rab7a	RAB7A, member RAS oncogene family	0.00020	4.42
Phagosome	Ncf4	neutrophil cytosolic factor 4	0.00020	4.42
Phagosome	Lamp1	lysosomal-associated membrane protein 1	0.00020	4.42
Phagosome	Fcgr2b	Fc fragment of IgG, low affinity IIb, receptor (CD32)	0.00020	4.42
Phagosome	Ctss	cathepsin S	0.00020	4.42
Phagosome	Cd209f	CD209f antigen	0.00020	4.42
Phagosome	Atp6v0e1	ATPase, H+ transporting, lysosomal, V0 subunit e1	0.00020	4.42
Valine, leucine and isoleucine degradation	Mccc2	methylcrotonoyl-CoA carboxylase 2 (beta)	0.00160	15.15
Valine, leucine and isoleucine degradation	Hmgcs1	3-hydroxy-3-methylglutaryl-CoA synthase 1 (soluble)	0.00160	15.15
Valine, leucine and isoleucine degradation	Hibch	3-hydroxyisobutyryl-CoA hydrolase	0.00160	15.15
Valine, leucine and isoleucine degradation	Ehhadh	enoyl-CoA, hydratase/3-hydroxyacyl CoA dehydrogenase	0.00160	15.15
Valine, leucine and isoleucine degradation	Aldh3a2	aldehyde dehydrogenase 3 family, member A2	0.00160	15.15

## Discussion

**Figure 5.**
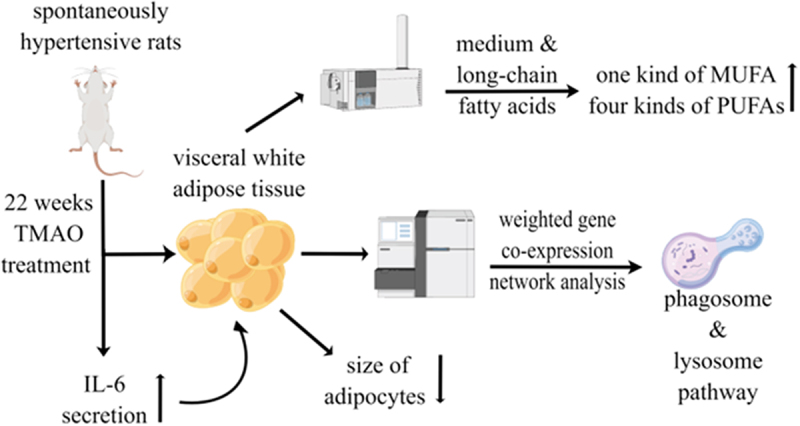
A summary figure showing the main results by Figdraw (www.figdraw.com).

An interesting finding of our study was the metabolic role of TMAO on the vWAT of SHRs. We found that TMAO could regulate the metabolic status of the vWAT of SHRs, including lipogenesis reduction and improvement of specific fatty acids composition. The mechanism underlying this effect was more likely to involve the regulation of phagosomes and lysosomes in vWAT (Figure 5).

Increased exposure to TMAO is associated with atherosclerosis and major adverse cardiovascular events. The TMAO pathway likely interacts with pathogenic mechanisms, including the development of atherosclerotic plaque iteration in macrophages [3,5,32] and the promotion of platelet hyper-responsiveness [33]. Furthermore, circulating TMAO induces low-grade chronic systemic inflammation [34] and increases levels of inflammatory cytokines by promoting NF-κB activation [35] and activating the NLRP3 inflammasome [26]. Moreover, recent studies have revealed that TMAO was positively associated with cardio-metabolic risk factors, such as insulin resistance and metabolic syndrome.

Consequently, we measured six inflammatory serum cytokines, IL-6, IL-1β, TNF-α, MCP-1, insulin, and leptin, to ascertain the effects of chronic treatment with TMAO on vWAT in SHRs. Notably, although no overt inflammation was observed, elevated circulating levels of IL-6 and insulin might reveal a possible link between TMAO and vWAT. Novel beneficial roles for TMAO in glucose homoeostasis and insulin secretion have been reported, and the improvement of impaired glucose tolerance mediated by increased insulinemia was related to ER-stress improvement, which was confirmed in vitro by treating isolated pancreatic islets with subcutaneous TMAO infusion [36]. Consistently, our results showed that chronic high-dose TMAO treatments triggered insulin secretion, which might activate hepatic lipogenesis [37]. Although the circulating levels of IL-6 were elevated by the TMAO treatment in this study, other inflammation-associated cytokines, IL-1β, TNF-α, and MCP-1, did not show statistical differences. In addition, IL-6 has context-dependent pro- and anti-inflammatory properties [27]. Therefore, the inflammatory responses induced by TMAO might still not be confirmed. However, clear evidence has shown that IL-6 and insulin can regulate adipose tissue, but that they had opposite effects. On the one hand, IL-6 induces adipocyte lipolysis via activating the STAT3 pathway [38], which is associated with anti-inflammatory signalling [39]; however, on the other hand, insulin plays an important role in inhibiting lipolysis [40].

To evaluate the effects of elevated levels of IL-6 and insulin on vWAT mediated by TMAO, we observed the morphology of vWAT through HE and Masson staining. The beneficial effects of a 22-week TMAO treatment resulted in adipocyte volume diminishment through increased IL-6 secretion [36]. Thus, compared with the effect of lipogenesis stimulated by insulin, the effect of IL-6 on lipolysis occupied the dominant role during TMAO treatment in SHRs.

Combined with our previous results and the well-known stimulatory effect of IL-6 on lipolysis, as well as fatty acid oxidation [31], TMAO was highly likely to regulate the fatty acid composition in vWAT. Therefore, a gas chromatography-tandem mass spectrometer analysis was performed to determine the changes in the composition of fatty acids. In this study, we found that TMAO elevated one MUFA (cis-15-tetracosenoate) and four PUFAs [cis-11,14,17-eicosatrienoic acid, docosatetraenoate, docosapentaenoate n-3 (C22:5N3), and docosapentaenoate n-6 (C22:5N6)] in vWAT. Adherence to a balanced diet may help postpone the onset of cardiovascular disease (CVD) and extend life. These diets were mostly composed of fatty acids [41]. The beneficial effects of MUFA and PUFA for CVD may alleviate systolic and diastolic blood pressure by alleviating oxidative stress and modifying the activities of membrane-associated proteins [42,43]. Cis-15-tetracosenoic acid, also called nervonic acid, was increased in vWAT by the TMAO treatment. It is a long-chain fatty acid with significant biological functions, including increasing brain progression, enhancing memory, and delaying brain ageing [44]. A study examining the relationship between erythrocyte omega-9 monounsaturated fatty acids and all-cause and cardiovascular mortality discovered that cis-15-tetracosenoic acid is an independent predictor of cardiovascular death [45]. For PUFAs, many health benefits have been reported. For example, marine n-3 PUFAs might protect against acute coronary syndrome in men [46], and increased n-6 PUFA consumption is related to a decreased risk of CVD [47]. However, prospective observational studies may support the primary prevention of atherosclerotic cardiovascular disease with PUFAs, whereas randomized controlled studies frequently yielded neutral conclusions [48]. Because the health effects of PUFAs might be influenced by their metabolic key enzymes [49], further investigations should focus on individual differences in those enzymes’ activity levels. Our results disclosed that TMAO was able to increase two n-3 PUFAs (cis-11,14,17-eicosatrienoic acid and docosapentaenoate n-3) and two n-6 PUFAs (docosatetraenoate and docosapentaenoate n-6). Eicosatrienoic acid, an elongation product of polylactic acid, shows anti-inflammatory properties through nuclear factor kappa B activity downregulation [50–52]. Docosapentaenoic acid has been frequently overlooked in lipid research. Although a previous study demonstrated that docosapentaenoic n-3 and docosapentaenoic n-6 enhances lipoprotein profiles and aortic function in hamsters fed a high cholesterol diet [53], the true functionality of these PUFAs remains obscure until more is known regarding the properties of unique, specialized pro-resolving lipid mediators [54].

To further investigate the potential mechanism of TMAO-mediated vWAT regulation, we performed whole transcriptome sequencing and a comprehensive bioinformatics analysis. WGCNA, a powerful tool for identifying key functional modules in big data analysis [25,55], was employed to undertake a systems-level view of transcriptional differences associated with the TMAO treatment in SHRs.

The results of the WGCNA indicated that TMAO mainly regulates metabolic pathways in vWAT. Interestingly, the fatty acid metabolism pathway was also identified as a most significantly altered biological pathway, which supported our results that TMAO could regulate the composition of fatty acids in SHRs. Our results also showed that the expression of *Ehhadh* and *Aldh3a2*, which are associated with TMAO treatments in SHRs, function in both fatty acid metabolism and valine, leucine, and isoleucine degradation pathways. *EHHADH* encoded a bifunctional beta-oxidation enzyme involved in peroxisomal fatty acid oxidation [56]. *ALDH3A2* is a classic target gene of PPARα, being involved in peroxisomal and mitochondrial fatty acid oxidation [57].

In addition, phagosome and lysosome pathways, which are closely associated with fatty acid metabolism, were also enriched in the WGCNA. Additionally, *Lamp1* and *Ctss* were found in the same co-expression module, which was strongly associated with TMAO therapy. They play roles in both phagosome and lysosome pathways [58]. The potential mechanism of TMAO regulating phagosome and lysosome pathways might be associated with its effect of reducing the endoplasmic reticulum-stress response [36,59]. Lipolysis and autophagy share similar regulatory mechanisms. When nutrients are scarce for the generation of energy, cellular lipids in lipid droplets are degraded into fatty acids. Autophagy induction is a cellular response to the famine that occurs for the second time, and it transports intracellular proteins and organelles sequestered in autophagosomes to lysosomes for degradation and energy production [60]. In addition, SFAs prevent autophagosome-lysosome fusion. As a result, intracellular protein aggregates develop, while enhancing the breakdown of autophagic vacuoles, and unsaturated fatty acids counteract the effects of SFAs [61].

These results indicated that TMAO could shift metabolic pathways, which was associated with IL-6 secretion, especially those related to fatty acid metabolism, and the metabolic alterations of vWAT may be implicated in phagosome and lysosome pathways.

Several limitations of our study should be acknowledged. First, this study focused on both long-chain and medium-chain fatty acids. However, an abundance of research demonstrated that short-chain fatty acids are also critical for health maintenance and disease progression [62], but further investigations are required. Second, although the WGCNA of transcriptome sequencing indicated the potential mechanisms of fatty acid metabolic regulation via TMAO treatment in vWAT, experimental experiments remain required to determine such mechanisms. Third, further research is required to explore the effects of insulin induced by TMAO on vWAT in SHRs. Last, TMAO treated WYKs and high-fat diet-fed SHRs also need to be investigated because these experiments could reveal important information about the effects of TMAO on vWAT.

## Conclusion

The study results shed new light on the metabolic roles of TMAO on vWAT in SHRs. TMAO could regulate the metabolic status of the vWAT of SHRs, including lipogenesis reduction and improvement of specific fatty acid composition. The mechanism underlying this effect likely involves the phagosome and lysosome pathways in vWAT.

## Supplementary Material

Supplemental Material

## Data Availability

The datasets generated and/or analysed during the current study are available in the GEO database. (https://www.ncbi.nlm.nih.gov/geo/query/acc.cgi?acc= GSE188336).
