# Supplementary material for: The effect of trimethylamine N-oxide on the metabolism of visceral white adipose tissue in spontaneously hypertensive rat
Source: Adipocyte. 2022 Aug 17;11(1):420–33. doi: 10.1080/21623945.2022.2104783 (PMC9387326; doi:10.1080/21623945.2022.2104783)
Supplement: Supplemental Material [file KADI_A_2104783_SM4409.zip › Supplementary/Table S1.docx]

| Name | Linear equation | R^2 | Linear range (ug/ml) |
| --- | --- | --- | --- |
| C4:0 | y = 0.676115 * x+ 0.010459 | 0.999579272 | 0.002-50 |
| C6:0 | y = 2.145062 * x+ 2.400182E-004 | 0.999673401 | 0.002-50 |
| C8:0 | y = 2.808091 * x+ 3.552830E-004 | 0.999690439 | 0.002-50 |
| C10:0 | y = 2.974875 * x+ 4.407884E-004 | 0.999709411 | 0.004-100 |
| C11:0 | y = 2.938498 * x+ 3.125274E-004 | 0.999767058 | 0.002-50 |
| C12:0 | y = 2.924615 * x+ 3.922994E-004 | 0.999828801 | 0.004-100 |
| C13:0 | y = 2.774600 * x+ 3.058208E-004 | 0.999713304 | 0.002-50 |
| C14:0 | y = 2.578346 * x+ 0.015441 | 0.999618099 | 0.002-50 |
| C14:1N5 | y = 1.093605 * x- 6.418088E-004 | 0.999330953 | 0.002-50 |
| C15:0 | y = 2.534515 * x+ 1.383307E-004 | 0.999747865 | 0.002-50 |
| C15:1N5 | y = 0.979738 * x+ 1.153024E-004 | 0.999792521 | 0.002-50 |
| C16:0 | y = 2.542880 * x+ 0.071060 | 0.999665542 | 0.004-100 |
| C16:1N7 | y = 0.765158 * x+ 0.005247 | 0.999702007 | 0.002-50 |
| C17:0 | y = 2.399196 * x- 8.808896E-004 | 0.99969776 | 0.004-100 |
| C17:1N7 | y = 0.746499 * x+ 1.371566E-004 | 0.999695696 | 0.002-50 |
| C18:0 | y = 2.435727 * x+ 0.084298 | 0.999631009 | 0.004-100 |
| C18:1TN9 | y = 0.775054 * x- 0.004505 | 0.999647376 | 0.002-50 |
| C18:1N9 | y = 0.716432 * x+ 0.100592 | 0.999527484 | 0.004-100 |
| C18:2TTN6 | y = 0.964511 * x- 0.020342 | 0.999577512 | 0.002-50 |
| C18:2N6 | y = 0.956557 * x- 0.026392 | 0.999563221 | 0.002-50 |
| C18:3N6 | y = 0.781160 * x+ 3.118248E-004 | 0.999855424 | 0.004-100 |
| C18:3N3 | y = 1.046795 * x- 0.004403 | 0.999779978 | 0.002-50 |
| C20:0 | y = 1.870038 * x+ 1.526439E-004 | 0.999768281 | 0.004-100 |
| C20:1N9 | y = 0.827732 * x- 0.007419 | 0.999985157 | 0.002-50 |
| C20:2N6 | y = 0.914886 * x- 0.023660 | 0.999542002 | 0.002-50 |
| C21:0 | y = 1.552001 * x+ 0.001375 | 0.999867097 | 0.002-50 |
| C20:3N6 | y = 0.878719 * x- 0.029676 | 0.999492438 | 0.002-50 |
| C20:4N6 | y = 0.948662 * x- 0.047859 | 0.999535526 | 0.002-50 |
| C20:3N3 | y = 1.123874 * x- 0.064862 | 0.999479333 | 0.002-50 |
| C22:0 | y = 1.602850 * x- 2.476369E-004 | 0.999544385 | 0.004-100 |
| C20:5N3 | y = 1.080144 * x- 0.010530 | 0.999981046 | 0.002-50 |
| C22:1N9 | y = 0.940492 * x- 0.008849 | 0.999855724 | 0.002-50 |
| C22:2N6 | y = 0.548524 * x+ 3.968627E-005 | 0.999440545 | 0.002-50 |
| C23:0 | y = 1.880202 * x- 0.008220 | 0.999889805 | 0.002-50 |
| C22:4N6 | y = 1.009597 * x- 0.025842 | 0.999199063 | 0.002-50 |
| C22:5N6 | y = 1.021545 * x- 0.025451 | 0.999221472 | 0.002-50 |
| C24:0 | y = 1.786569 * x- 0.018456 | 0.999835667 | 0.004-100 |
| C22:5N3 | y = 1.026703 * x- 0.038717 | 0.999941074 | 0.002-50 |
| C24:1N9 | y = 1.041510 * x- 0.048647 | 0.999675 | 0.002-50 |
| C22:6N3 | y = 1.096107 * x- 0.067056 | 0.999531592 | 0.002-50 |

Table S1
