# Supplementary figures and images for: The effect of trimethylamine N-oxide on the metabolism of visceral white adipose tissue in spontaneously hypertensive rat
Source: Adipocyte. 2022 Aug 17;11(1):420–33. doi: 10.1080/21623945.2022.2104783 (PMC9387326; doi:10.1080/21623945.2022.2104783)

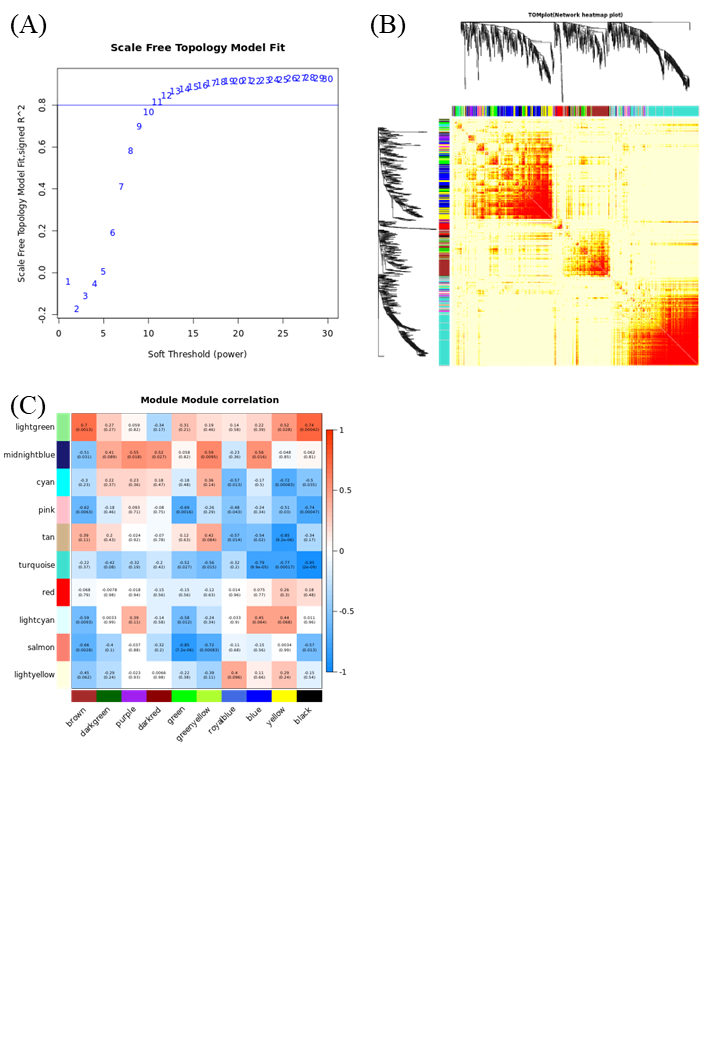

Supplement: Supplemental Material [file KADI_A_2104783_SM4409.zip › Supplementary/Figure S1.PNG]
